# Operationalizing transparency regarding the use of health data for research: a cross-sectional survey of citizens’ perspectives

**DOI:** 10.1186/s12889-026-28232-0

**Published:** 2026-07-08

**Authors:** Annabelle Cumyn, Jean-Frédéric Ménard, Emmanuel Bilodeau, Roxanne Dault, Jean-Philippe Morissette, Marie-Pier Domingue, Félix Camirand Lemyre, Denis Boutin, Jean-François Ethier

**Affiliations:** 1 Groupe de recherche interdisciplinaire en informatique de la santé, Faculté de médecine et des sciences de la santé, Université de Sherbrooke, 3001 12e Avenue Nord, Sherbrooke, Québec J1H 5H3 Canada; 2 Département de médecine, Faculté de médecine et des sciences de la santé, Université de Sherbrooke, Sherbrooke, Québec Canada; 3 Faculté de droit, Université de Sherbrooke, Sherbrooke, Québec Canada; 4 Département de philosophie et d’éthique appliquée, Faculté des lettres et sciences humaines, Université de Sherbrooke, Sherbrooke, Québec Canada; 5 Département de mathématiques, Faculté des sciences, Université de Sherbrooke, Sherbrooke, Québec Canada

**Keywords:** Public opinion, Transparency mechanisms, Health data, Secondary use, Survey

## Abstract

**Background:**

Secondary use of health data can accelerate discovery and enable personalized care, yet social acceptability relies on autonomy, transparency, and trust, especially in the context of security breaches and concerns about misuse. In Quebec, as in many Western settings, there is no formal mechanism to inform individuals about such use, underscoring the need for practical solutions. This quantitative cross-sectional study assessed whether transparency mechanisms increase trust in the use of health data for research; examined their perceived priority and acceptability; and explored effects across population subgroups.

**Methods:**

We conducted a cross-sectional telephone survey among 366 adults in Quebec (Canada) selected via stratified random sampling. The sample included a random selection of adult household members based on age. Descriptive statistics, chi-square tests, and exploratory multivariate logistic regressions were performed.

**Results:**

More than half of respondents were unaware that their health data may be used for research. Trust was higher for public-sector than private-sector researchers. Overall, 76% indicated that access to information would increase their trust, particularly younger adults (OR = 1.98, 95% CI: 1.10–3.57). Over 90% judged implementing transparency mechanisms within five years as important, with stronger support among those aware of current data use (OR = 4.63, 95% CI: 1.33–16.20). A secure website was considered acceptable by 75% of respondents as a transparency mechanism. Individuals with high trust in public-sector researchers (OR: 2.06, 95% CI: 1.08–3.94) and younger adults (OR: 2.05, 95% CI: 1.13–3.71) were more likely to consider it important. Exploratory analyses suggested that awareness of current data use for research and pre-existing trust predict increased trust in researchers from various public and private organizations if a secure website is implemented.

**Conclusion:**

Providing information through a transparency mechanism, particularly via a secure website, appears acceptable, valued, and potentially effective for increasing trust in the use of health data for research. Our findings support the feasibility of a web-based transparency portal and suggest tailoring communication to demographic, trust and awareness profiles.

**Supplementary Information:**

The online version contains supplementary material available at https://doi.org/10.1186/s12889-026-28232-0.

## Background

Secondary use of health data collected during patient care has an established potential to increase the speed of scientific discovery and implement personalized health care [1–3]. From the patient’s perspectives, there are many concurrent conversations around data sharing and its secondary use soliciting different ethical values such as privacy, autonomy, reciprocity, altruism, etc. [4–8]. While some data sharers emphasize the importance of data altruism as a contribution to the common good, citizens in our recent survey prioritized autonomy when asked : *“Is it more important that each person may easily decide the use or not of their medical data in research OR that the people’s medical data be easily accessible in research?”* [9]. This importance given to autonomy over common good occurs in a context where numerous studies have shown the importance of building and maintaining public trust [10, 11]. Given significant events of security breaches and data misuse, and a potential deficit in trust, more attention might be given to the risks associated with data sharing rather than the risks of not using the data to improve knowledge and care [12–18].

Given this natural focus on autonomy and an expectation of having some control over one’s data, the right path to sustained social acceptability over heath data reuse has yet to be defined. We posit that knowledge and understanding will be essential factors to support trust (and informed consent where applicable), and respect citizen expectations regarding transparency. This double objective is important as illustrated in a previous survey in which citizens were more inclined to allow secondary use of their data when transparency is there, even when consent is not possible [9].

In Quebec, as in many Western jurisdictions, there is no formal, automated mechanism to inform individuals about the use of their own health data for research purposes. The primary objective of this study was to assess whether providing access to information, through a transparency mechanism, influences trust in the use of health data for research purposes. The secondary objectives focused on how to operationalize this transparency, specifically by examining whether (a) implementing an information-sharing tool should be prioritized within the next five years at the national level, and (b) whether a secure website would serve as an effective method of providing information for the population. An additional exploratory objective was to assess whether such website would increase trust in researchers and for which subgroups of the population.

## Methods

### Study design and participants

We conducted a cross-sectional telephone survey among French- and English-speaking adults in Quebec selected via stratified random sampling based on age. Descriptive statistics, chi-square tests, and exploratory multivariate logistic regressions were performed. Sociodemographic characteristics collected included age, level of education and health care utilization in the past year.

This survey was conducted as part of a mixed methods study whose aim is to identify more broadly the information to be communicated to the public about research carried out using health data, and the methods for communicating this information.

### Participant recruitment and selection

Survey administration was managed by a specialized survey firm [19]. Participants were selected randomly after confirming a valid telephone number, using a two-step process: (1) A non-proportional stratified random sampling of households in three strata (Montreal, Quebec City, and the rest of the province of Quebec), (2) a random selection of an adult household member based on age.

### Sample size calculation

The sample size for this survey was determined before data collection based on the following main survey question: “Ways could be put in place to inform you about how your health data is used for research. If you had access to information, would it increase your trust in the use of your health data for research?”. A sample size of 365 participants provided sufficient precision to detect a statistically significant difference from p0 = 50% using a 95% Wald-type confidence interval for a sample proportion, with a margin of error of approximately 5.1%.

### Survey questionnaire

The survey content was developed in an iterative process by our research team in close collaboration with a research patient-partner (D.B.) and an organization accompanying citizens with lower literacy levels. Questions identified in the survey were based on findings from two scoping review articles previously conducted by our research team on transparency’s impact and public expectations of transparency in the context of health data secondary use [20, 21].

Initially designed in French, the questionnaire was translated in English and validated by an English-speaking plain language expert. The detailed questionnaire is provided in Supplementary Material 1. In the questionnaire and throughout the manuscript, each survey question is labeled as [QX], where X represents a keyword describing the question. Sociodemographic questions are labeled as [SQX].

The survey was initially pre-tested with 10 participants. A team member (R.D.) reviewed recorded interviews to ensure clarity and provide feedback to interviewers. Experienced interviewers then conducted the computer-assisted survey, which was administered to members of the public over a 3-week period between October 25 and November 15, 2023.

### Study outcome questions

To address the primary objective of this study, we used the following survey question as the outcome measure in our analyses:

- *Primary outcome. “If you had access to information*,* would it increase your trust in the use of your health data for research?”* [Qinfo-trust]

To address our secondary objectives, we used the following survey questions as outcome measures in our analyses:

- *Secondary outcome (1) “In the next 5 years*,* would it be important for Quebec citizens to have access to ways to inform them about how their health data is used for research?”* [Qinfo-priority]
- *Secondary outcome (2) “In your opinion*,* would a secure website be a good way to inform you about how your health data is used for research?”* [Qinfo-website]

Finally, to explore whether a website would increase trust in researchers from different organizations, we used the following survey questions as outcome measures in our analyses:

- *“Would such a secure website increase your level of trust in researchers from:”*
  - *◦ Exploratory outcome (1) “Universities?”* [Qinctrust-uni]
  - *◦ Exploratory outcome (2) “Government-related organizations?”* [Qinctrust-gov]
  - *◦ Exploratory outcome (3) “The healthcare system?”* [Qinctrust-health]
  - *◦ Exploratory outcome (4) “Private companies based in Quebec?”* [Qinctrust-privQc]
  - *◦ Exploratory outcome (5) “Private compagnies based outside Quebec?”* [Qinctrust-privOut]

We used every item as a distinct outcome to identify potential factors of interest related to every specific type of organization and provide better guidance to stakeholders.

### Predictors

The choice of predictors for the analyses was made a priori (before accessing the raw data) by the research team based on expert inputs and prior literature [20, 21]. The predictors are derived from survey questions spanning three main dimensions, as detailed below. This section describes these survey questions and how the predictors were generated from them. Variables derived from survey questions are referred to as [VarX] to differentiate them from the raw survey questions denoted [QX], which can be found in Supplementary Material 1.

### Respondents’ knowledge of the use of health data for research in Quebec

The predictor [VarKnow] is based on two survey questions [Qknow1; Qknow2] about respondents’ knowledge of the use of health data for research in Quebec. These questions were asked at the beginning of the survey, before giving respondents more information about the current regulatory model for data access. This methodological choice was made to avoid influencing the measure of baseline knowledge and to capture their actual knowledge on the matter. Following these two questions, the survey included a paragraph informing respondents that currently, in Quebec, health data may be used by researchers without their knowledge or consent, so that each respondent would answer all other survey questions on equal footing.

The three categories generated for the predictor [VarKnow] are:

- Category 1 (No at [Qknow1]): Individuals who don’t think that their health data is being used for research *(reference category);*
- Category 2 (Yes at [Qknow1] and No at [Qknow2]): Individuals who think that their health data is being used for research, but not without their knowledge;
- Category 3 (Yes at [Qknow1] and Yes at [Qknow2]): Individuals who think that their health data is being used for research, and without their knowledge (current situation in Quebec).

Categories 1 and 2 reflect respondents who held incorrect initial knowledge. In Quebec, observational health studies are permitted to access health data (often deidentified, but not always) without having to inform the individuals concerned, which corresponds to category 3. In practice, two indicator variables were created to integrate this predictor into the analyses. Whenever a respondent preferred not to answer either [Qknow1] or [Qknow2], both values of the indicator variables were set as missing values.

### Respondents’ trust in researchers from different types of organizations

- To favor both model parsimony and interpretability, we considered regrouping questions into interpretable latent variables, thereby reducing the dimension of predictors. The questions pertaining to the respondents’ baseline level of trust in researchers from different types of organizations, i.e. [Qpretrust-uni; Qpretrust-gov; Qpretrust-health; Qpretrust-privQc; Qpretrust-privOut], were deemed most appropriate to be potentially regrouped as interpretable latent variables. As such, the following scenarios representing various combinations of the trust-related questions above were considered based on experts’ opinion. The scenarios were elaborated to ensure relevant interpretability of the variables obtained for the regression model and to cover possible correlation structures that could be encountered. Scenario A (5 variables – Dimensionality is not reduced). Trust in the five types of organizations [Qpretrust-uni; Qpretrust-gov; Qpretrust-health; Qpretrust-privQc; Qpretrust-privOut] has a minor correlation structure. Trust in each organization should be considered on its own and be used as distinct predictors;
- Scenario B (2 variables – Dimensionality is reduced). Trust towards the three types of public organizations [Qpretrust-uni; Qpretrust-gov; Qpretrust-health] is highly correlated. A single variable combining this information could be used as a predictor [VarTrust-Pub]. Also, trust in the two types of private organizations [Qpretrust-privQc; Qpretrust-privOut] is highly correlated. A single variable combining this information could be used as a predictor [VarTrust-Priv];
- Scenario C (1 variable – Dimensionality is reduced). Trust in the five types of organizations is highly correlated. A single variable combining this information could be used as a single predictor [VarTrust-All].

To determine which of the three scenarios should be retained, we conducted analyses based on the variance-covariance matrix of respondents’ answers to the question described above. Details on the creation of these variables and the handling of missing data are provided in Supplementary Material 2. In addition, because all five trust-related questions were measured on a four-item Likert scale (*very low trust*,* low*,* high*,* and very high*), the trust-related variables derived from them were dichotomized into two categories, depending on the results of the previous steps, to ensure interpretability of their coefficient estimates when used as predictors in the regression analyses:

- Category (1) Low level of trust *(reference category);*
- Category (2) High level of trust.

Details on how these categories were derived from the survey responses are provided in Supplementary Material 2.

### Respondents’ sociodemographic characteristics

For the sake of model parsimony and for the purpose of this analysis, the predictors based on sociodemographic characteristics, i.e. age [SQage], level of education [SQedu] and health care utilization in the past year [SQhcu], were dichotomized in a way that ensures comparability with previous surveys conducted on similar populations [9]. These three variables were therefore derived as follows:

- Age [VarAge]
  - ◦ Category (1) 18–44 years old;
  - ◦ Category (2) 45 years old or more *(reference category).*
- Level of education [VarEdu]
  - ◦ Category (1) No degree, high school degree or vocational degree *(reference category);*
  - ◦ Category (2) College or university degree.
- Health care utilization in the past year [VarHcu]
  - ◦ Category (1) Light user (5 visits or less) *(reference category);*
  - ◦ Category (2) Heavy user (6 visits or more).

Whenever a respondent preferred not to answer a question related to a given sociodemographic characteristic, the associated indicator variable was assigned a missing value.

### Statistical analyses

Statistical analyses were performed using *R 4.5.0 (R Core team*,* released on 2025-04-11)* [22].

### Descriptive analyses

Descriptive analyses (proportions, percentages and count tables) were applied to report respondents’ answers to questions used for predictors derivation and for predictors themselves, as well as for the following additional sociodemographic characteristics: gender, language most often spoken at home, minority groups (First Nations and visible minorities), regular internet access and previous or current participation in health research studies.

### Tests on proportions

To assess the primary and secondary objectives, two-sided chi-squared tests for proportions were performed at a nominal significance level of 5% (no adjustment for multiplicity was undertaken). Corresponding confidence intervals (CI) were reported to see whether they include, or not, the baseline proportion of 50%.

### Exploratory regression analyses

Firstly, exploratory multivariate analyses were conducted using logistic regression models to examine associations between derived predictors and the primary and secondary study outcomes. Secondly, to examine the situation where there would be access to a website providing information regarding the use of health data, the same analyses were conducted to assess the association between the predictors and increased trust in researchers from different types of organizations, i.e. exploratory outcomes. Predictors included in the regression models were identified a priori by the research team, as described in methods section *Predictors*. Details on the models are presented in Supplementary Material 2. The statistical significance of each predictor variable was assessed at a nominal two-sided significance level of 5%. Corresponding CI of odds ratios (OR) are presented through forest plots.

In an effort to avoid introducing bias, multiple imputations by chained equations were used before creating the regression models to account for missing data, assuming data were missing at random [23]. Study outcomes [Qinfo-trust, Qinfo-priority, Qinfo-website, Qinctrust-uni, Qinctrust-gov, Qinctrust-health, Qinctrust-privQc, Qinctrust-privOut] as well as derived predictors [VarKnow, VarAge, VarEdu, VarHcu, VarTrust-Pub, VarTrust-Priv] were all included in the imputation model and were also imputed through logistic regressions.

Sensitivity analyses were conducted to, (a) compare the results obtained from the multiple‑imputation datasets with those from a complete‑case analysis, and (b) assess whether the trust‑related variables acted as mediators between the other predictors and the outcomes. Additional details are provided in Supplementary Material 2.

## Results

We first present the raw survey results, followed by the results based on the derived variables.

### Respondent characteristics

The survey included 366 members of the public. When data were available, the characteristics of the survey sample were compared with those of the broader Quebec population (Table 1).

**Table 1:** Respondents’ characteristics vs. Quebec population

	Survey *n*/*N* (%)	Quebec population*
Gender^+^		
Available data	363 / 366 (99.2%)	-
Male	157 / 363 (43.3%)	49.2%
Female	205 / 363 (56.5%)	50.5%
Transgender or non-binary	1 / 363 (0.3%)	0.2%
Missing information	3 / 366 (0.8%)	-
Age^¥^		
Available data	364 / 366 (99.5%)	-
18–44 years old	110 / 364 (30.2%)	39.6%
45–64 years old	122 / 364 (33.5%)	34.1%
65 years old or more	132 / 364 (36.3%)	26.3%
Missing information	2 / 366 (0.5%)	-
Highest degree obtained^+^		
Available data	359 / 366 (98.1%)	-
No degree	24 / 359 (6.7%)	18.2%
High school or vocational degree	96 / 359 (26.7%)	37.2%
College or University degree	239 / 359 (66.6%)	44.6%
Missing information	7 / 366 (1.9%)	-
Language most often spoken at home		
Available data	365 / 366 (99.7%)	-
French	325 / 365 (89.0%)	78.9%
English	24 / 365 (6.6%)	10.6%
Other	16 / 365 (4.4%)	10.5%
Missing information	1 / 366 (0.3%)	-
Number of medical visits in the last year		
Available data	359 / 366 (98.1%)	-
0–5 visits	220 / 359 (61.3%)	-
6 visits or more	139 / 359 (38.7%)	-
Missing information	7 / 366 (1.9%)	-
First Nations		
Available data	358 / 366 (97.8%)	-
Yes	13 / 358 (3.6%)	2.5%
Missing information	8 / 366 (2.2%)	-
Visible minority		
Available data	361 / 366 (98.6%)	-
Yes	32 / 361 (8.9%)	16.1%
Missing information	5 / 366 (1.4%)	-
Regular access to Internet^&^		
Available data	362 / 366 (98.9%)	-
Yes	329 / 362 (90.9%)	91.8%
Missing information	4 / 366 (1.1%)	-
Has previously participated in a health research project		
Available data	362 / 366 (98.9%)	-
Yes	55 / 362 (15.2%)	-
Missing information	4 / 366 (1.1%)	-

*Latest census data (2021) for the province of Quebec [24]

^¥^Since data is available from age 15 in the census data of 2021 and from age 18 in our sample, we only considered age starting from 20 years old in the census data

^+^Data is available from age 15 in the census data of 2021 and from age 18 in our sample

^&^Quebec population: Data of the *Institut de la statistique du Québec* of 2020 [25]

### Respondents’ answers to predictor questions

Respondents’ answers to the predictor survey questions are presented in Tables 2 and 3.

**Table 2:** Knowledge of the use of health data for research

	Available data *n*/*N* (%)	Yes *n*/*N* (%)	No *n*/*N* (%)
In Quebec, do you think your health data is used for research?	321 / 366 (87.7%)	149 / 321 (46.4%)	172 / 321 (53.6%)
*In Quebec, do you think your health data is used for research without your knowledge?	144 / 149 (96.6%)	88 / 144 (61.1%)	56 / 144 (38.9%)

*[Qknow2] was only asked to respondents who answered ‘yes’ to [Qknow1]

**Table 3:** Level of trust in researchers from different types of organizations

What level of trust do you have in researchers from…	Available data *n*/*N* (%)	Very high *n*/*N* (%)	High *n*/*N* (%)	Low *n*/*N* (%)	Very low *n*/*N* (%)
Universities?	364 / 366 (99.5%)	97 / 364 (26.6%)	206 / 364 (56.6%)	47 / 364 (12.9%)	14 / 364 (3.8%)
Government-related organizations?	364 / 366 (99.5%)	76 / 364 (20.9%)	205 / 364 (56.3%)	59 / 364 (16.2%)	24 / 364 (6.6%)
The healthcare system?	364 / 366 (99.5%)	94 / 364 (25.8%)	209 / 364 (57.4%)	50 / 364 (13.7%)	11 / 364 (3.0%)
Quebec private companies?	364 / 366 (99.5%)	48 / 364 (13.2%)	167 / 364 (45.9%)	119 / 364 (32.7%)	30 / 364 (8.2%)
Private companies based outside Quebec?	359 / 366 (98.1%)	26 / 359 (7.2%)	124 / 359 (34.5%)	149 / 359 (41.5%)	60 / 359 (16.7%)

### Variable derivation

The frequency distribution (counts and percentages) related to the five variables derived [VarKnow; VarTrust; VarAge; VarEdu; VarHcu] are presented in Tables 4, 5 and 6.

As mentioned in the Methods section, respondents were categorized into three knowledge statuses. Based on this new variable, we observe that more than half of respondents (54.4%) did not believe that their health data is used for research purposes in Quebec, while only 27.8% demonstrated an awareness of the current situation regarding the secondary use of their data.

**Table 4:** Knowledge status

Knowledge of the use of health data for research [VarKnow]	*n*/*N* (%)
Available data	316 / 366 (86.3%)
Don’t think that health data is being used *(reference category)*	172 / 316 (54.4%)
Think that health data is being used, but not without their knowledge	56 / 316 (17.7%)
Think that health data is being used, and without their knowledge (*current situation)*	88 / 316 (27.8%)
Missing information	50 / 366 (13.7%)

Regarding trust in different types of organizations, the correlation matrix of those variables did not support weak correlation structure between those variables, leading us to discard scenario A (five variables used as predictors, for trust towards each organization), as defined in the *Predictors* methods section. The exploratory factorial analysis results reinforced this and led to the final selection of Scenario B (two variables used as predictors, one combining trust towards public organizations and one combining trust towards private organizations). Full analyses results are provided in Supplementary Material 2.

Based on these results and as described in the *Predictors* methods section, trust levels for the three types of public organizations (Universities, hospitals and government-related organizations) were combined according to section *Trust profiles calculations* of Supplementary Material 2 to create low and high trust categories, which was also done for the two types of private organizations (private Quebec companies, private companies based outside Quebec). Using these derived variables, we observe that more than 80% of respondents report high trust in researchers from public organizations, compared with only 40% who report high trust in researchers from private organizations (Table 5).

**Table 5:** Level of trust in researchers from public and private organizations

Trust in researchers from:	*n*/*N* (%)
Public organizations^§^ [VarTrust-Pub]	
Available data	365 / 366 (99.7%)
Low level of trust (*reference category*)	64 / 365 (17.5%)
High level of trust	301 / 365 (82.5%)
Missing information	1 / 366 (0.3%)
Private organizations^&^ [VarTrust-Priv]	
Available data	365 /366 (99.7%)
Low level of trust *(reference category)*	218 / 365 (59.7%)
High level of trust	147 / 365 (40.3%)
Missing information	1 / 366 (0.3%)

^§^Public organizations: Universities, hospitals, government-related organizations

^&^Private organizations: Private Quebec companies and private companies based outside Quebec

The results of the derived variables for age, education level, and health care utilization in the past year are presented in Table 6. Nearly 70% of respondents are aged 45 years or older, one third have no degree, a high school degree, or a vocational degree, and slightly over 60% are light users (5 visits or less in the last year) of the health care system.

**Table 6:** Respondents’ sociodemographic characteristics (dichotomized)

Sociodemographic profile	*n*/*N* (%)
Age [VarAge]	
Available data	364 / 366 (99.5%)
18–44 years old	110 / 364 (30.2%)
45 years old or more *(reference category)*	254 / 364 (69.8%)
Missing information	2 / 366 (0.5%)
Level of education [VarEdu]	
Available data	359 / 366 (98.1%)
No degree, high school degree or vocational degree *(reference * *category)*	120 / 359 (33.4%)
College or University degree	239 / 359 (66.6%)
Missing information	7 / 366 (1.9%)
Health care utilization in the past year [VarHcu]	
Available data	359 / 366 (98.1%)
Light user *(reference category)*	220 / 359 (61.3%)
Heavy user	139 / 359 (38.7%)
Missing information	7 / 366 (1.9%)

### Analyses results

#### Main study outcome: impact of information on trust in the use of health data for research

Over 75% of respondents indicated that having access to information about how their health data is used for research would increase their trust in such practices. This proportion is statistically significantly different from 50% (76.2%, CI: [71.5% – 80.3%]) (Table 7).

**Table 7:** Impact of information on trust in the use of health data for research

Ways could be put in place to inform you about how your health data is used for research. If you had access to information, would it increase your trust in the use of your health data for research?	*n*/*N* (%)	[95% CI]
Available data	361 / 366 (98.6%)	
Yes	275 / 361 (76.2%)	[71.5%, 80.3%]
No	86 / 361 (23.8%)	[19.7%, 28.5%]
Missing information	5 / 366 (1.4%)	

**Fig. 1:**
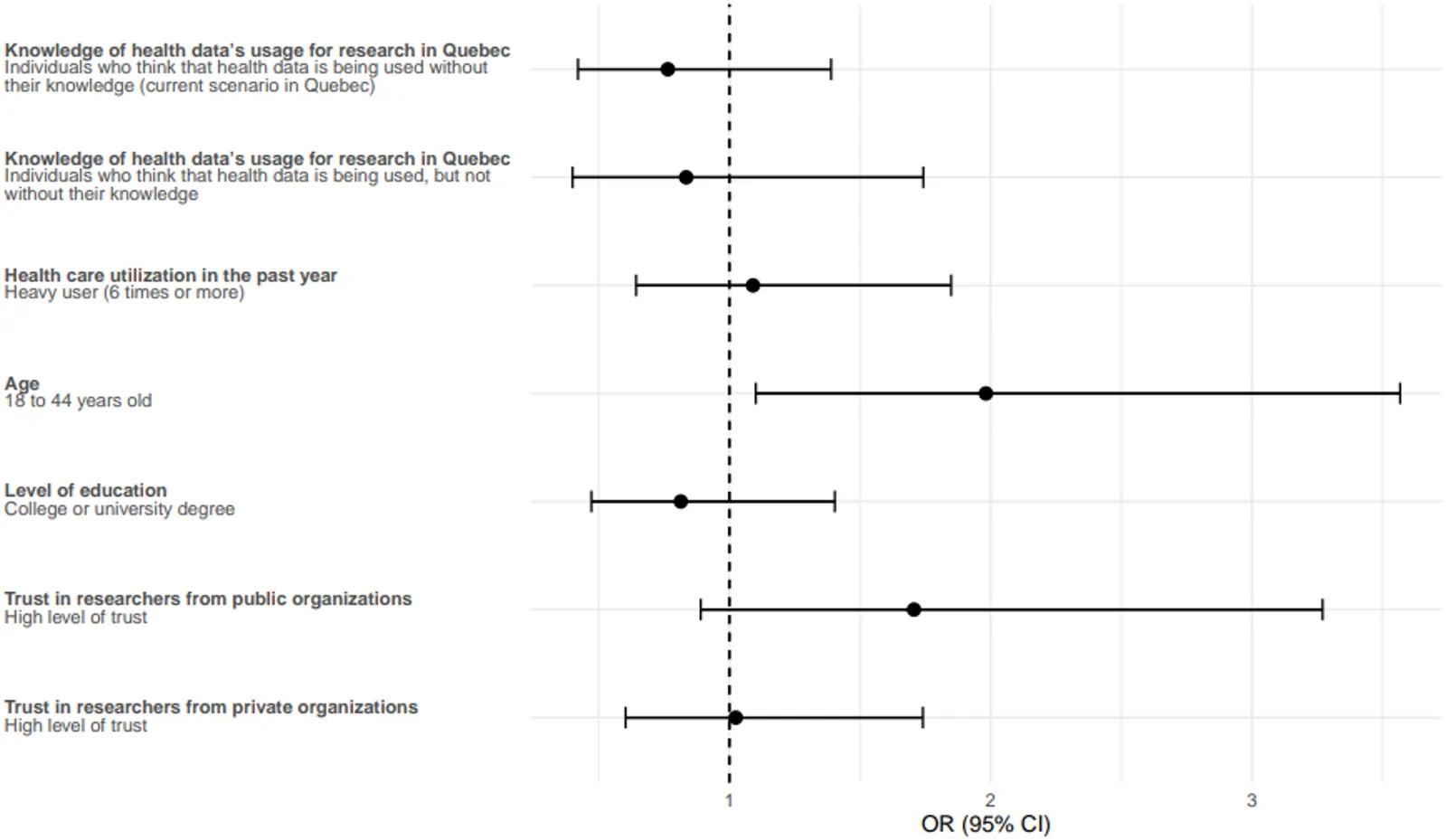
Predictors associated with an increased trust if being provided with information about the use of health data

According to the exploratory regression analysis (Fig. 1), age was the only predictor associated with this outcome. Individuals aged 18 to 44 were more likely to report increased trust if they are being provided with information about the use of health data, compared with those aged 45 or older (adjusted OR: 2.0, CI: [1.1–3.6]).

#### Secondary study outcome: priority for the implementation of transparency mechanisms

Over 90% of respondents believe it is important for Quebec’s population to have, within the next five years, ways to be informed about how their health data is used for research. This proportion is statistically significantly different from 50% (91.2%, CI: [87.9% – 93.7%]) (Table 8).

**Table 8:** Implementation of transparency mechanisms in the next five years

In the next 5 years, would it be important for Quebec citizens to have access to ways to inform them about how their health data is used for research?	*n*/*N* (%)	[95% CI]
Available data	365 / 366 (99.7%)	
Yes	333 / 365 (91.2%)	[87.9%, 93.7%]
No	32 / 365 (8.8%)	[6.3%, 12.1%]
Missing information	1 / 366 (0.3%)	

**Fig. 2:**
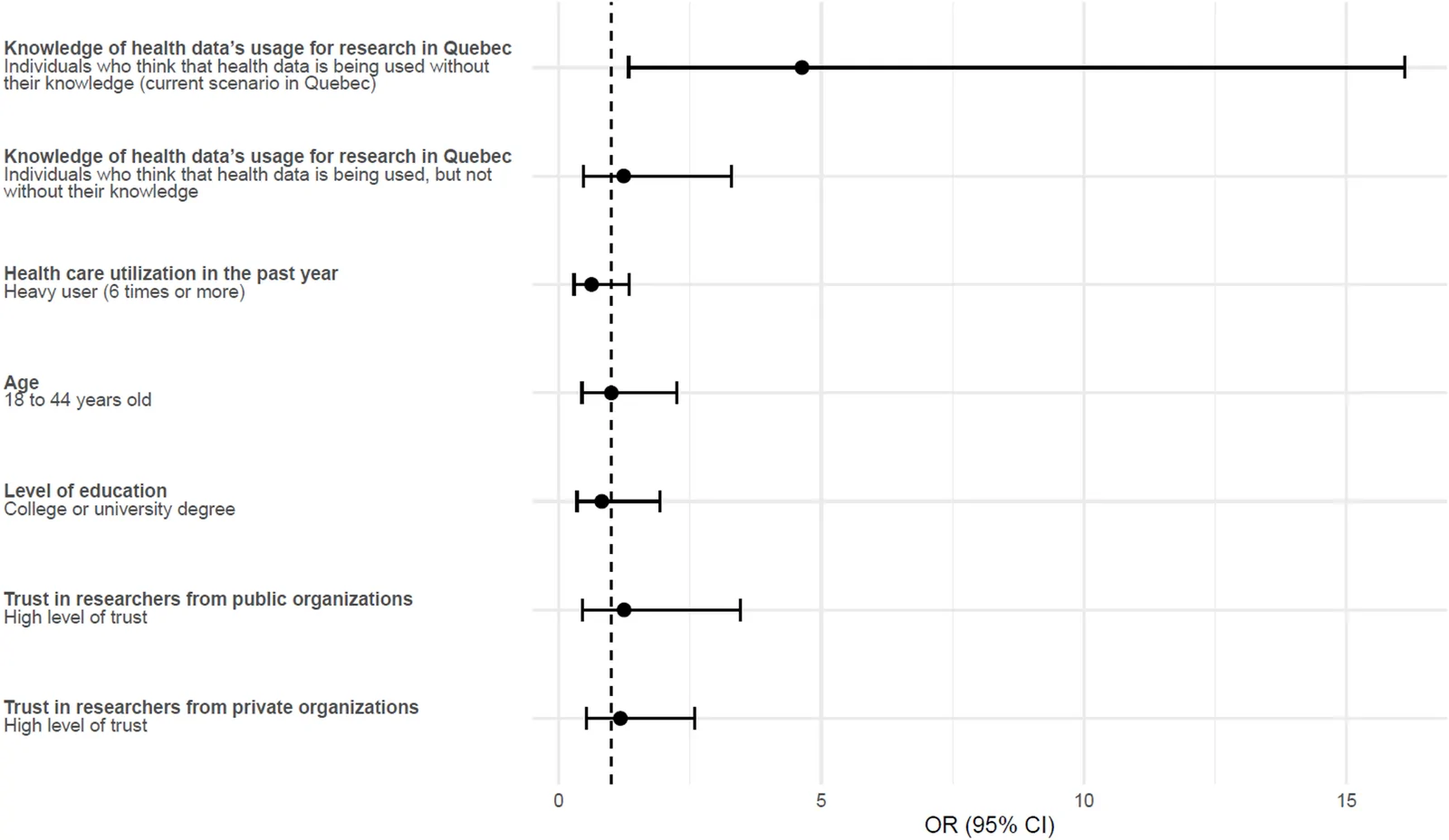
Predictors associated with the importance of having access to transparency mechanisms within the next five years

Individuals who are aware that health data is currently being used for research in Quebec without their knowledge (current scenario) are more likely to consider it important for the public to have access to ways of being informed about the use of their health data in the next five years, compared with individuals who do not believe their data is being used for research (adjusted OR: 4.6, CI: [1.3–16.2]) (Fig. 2).

#### Secondary outcome: a website as an acceptable method of communication

Over 75% of respondents consider a secure website to be an acceptable method for communicating information about how health data is used for research (Table 9). This proportion is statistically significantly different from 50% (75.3%, CI: [70.7% – 79.5%]).

**Table 9:** A website as a good method of communicating information about the use of health data for research

Would a secure website be a good way to inform you about how your health data is used for research?	*n*/*N* (%)	[95% CI]
Available data	365 / 366 (99.7%)	
Yes	275 / 365 (75.3%)	[70.7%, 79.5%]
No	90 / 365 (24.7%)	[20.5%, 29.3%]
Missing information	1 / 366 (0.3%)	

**Fig. 3:**
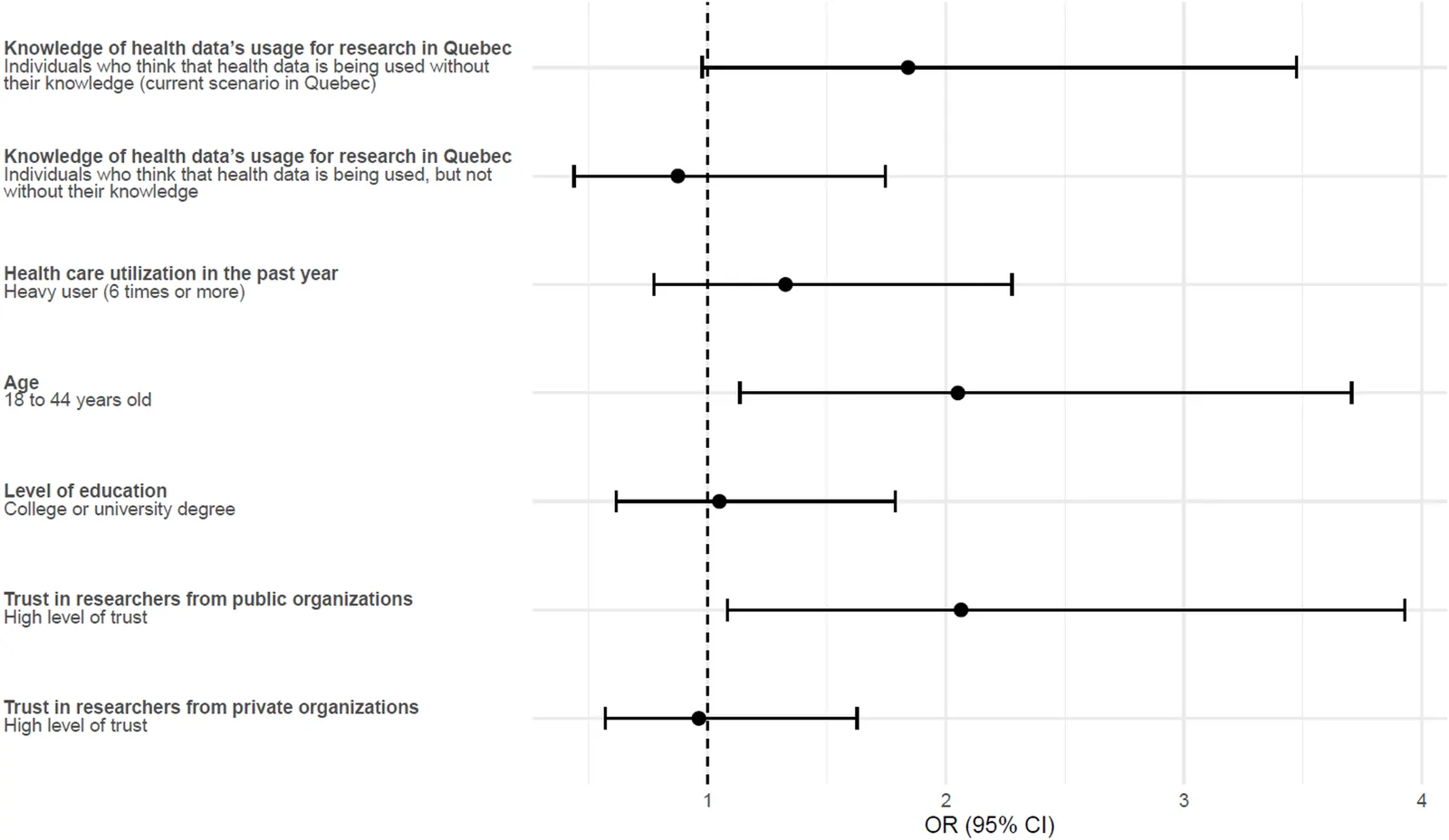
Predictors associated with finding a secure website an acceptable method of communicating information about the use of health data for research

Individuals with high trust in researchers from public organizations were more likely to consider a secure website an acceptable method for communicating information about the use of health data for research, compared with those with low trust (adjusted OR: 2.1, CI: [1.1–3.9]). Additionally, individuals aged 18 to 44 were more likely than those aged 45 or older to find this method of communication acceptable (adjusted OR: 2.1, CI: [1.1–3.7]) (Fig. 3).

#### Exploratory outcome: impact of a secure website on trust in researchers from different organizations

Between 68.3% and 74.7% of respondents indicated that their trust in researchers from different types of public organizations would increase if they had access to a secure website providing information on how their health data is used, with the strongest effect observed for researchers from the healthcare system (Table 10).

**Table 10:** Increased trust in researchers from public organizations with access to a secure website

Would a secure website increase your level of trust in researchers from…	Available data *n*/*N* (%)	Yes *n*/*N* (%)	No *n*/*N* (%)
Universities?	363 / 366 (99.2%)	253 / 363 (69.7%)	110 / 363 (30.3%)
Government-related organizations?	363 / 366 (99.2%)	248 / 363 (68.3%)	115 / 363 (31.7%)
The healthcare system?	363 / 366 (99.2%)	271 / 363 (74.7%)	92 / 363 (25.3%)

**Fig. 4:**
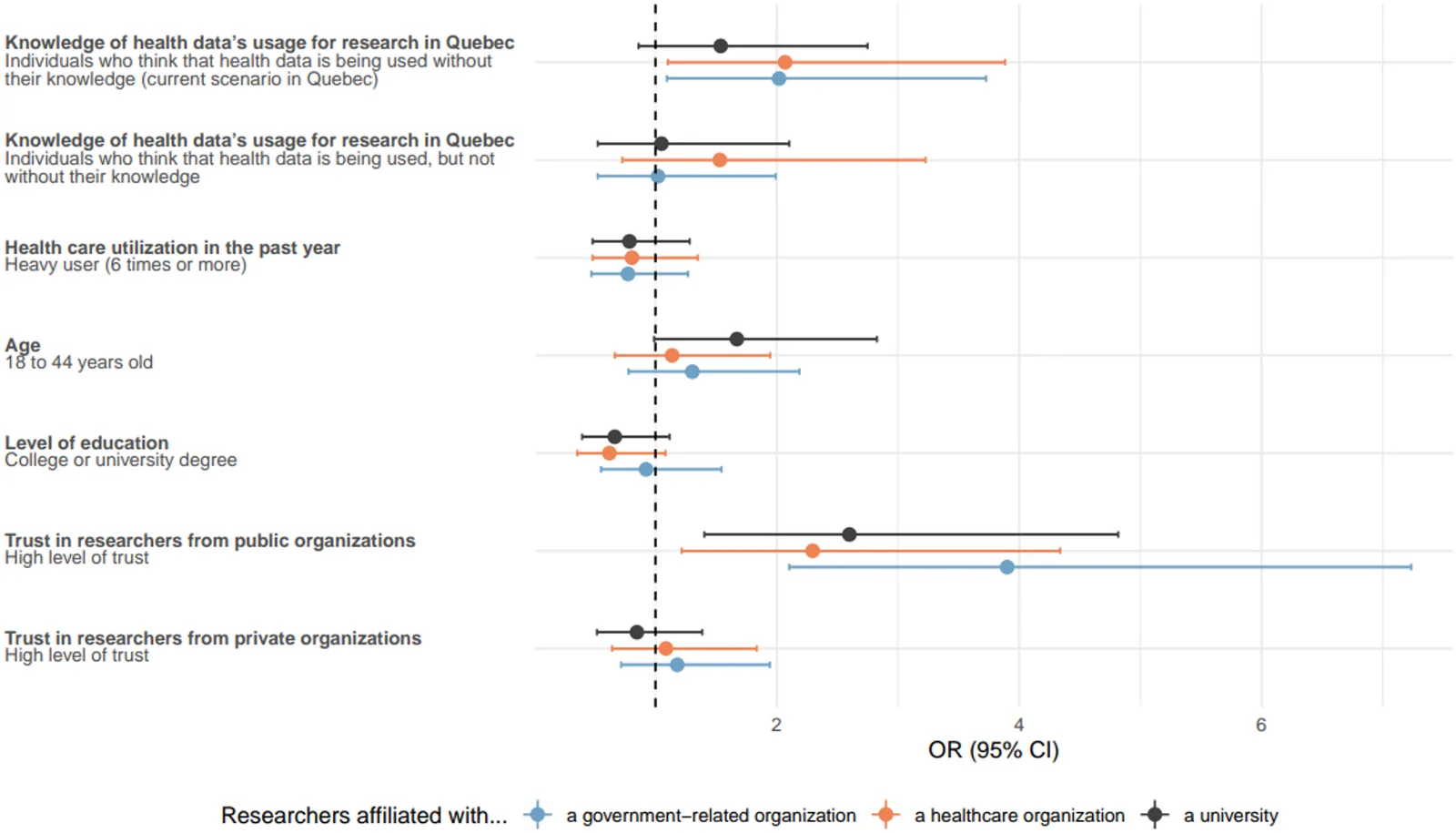
Predictors associated with increased trust in researchers from the three public organizations if a secure website is implemented

Individuals who are already aware that their health data is currently used for research in Quebec without their knowledge (referring to the existing data access model) were more likely to report increased trust in researchers from government-related or healthcare organizations if a secure website is implemented, compared with those who do not believe their data is currently used for research (government-related organizations: adjusted OR: 2.0, CI: [1.1–3.7]; healthcare organizations: adjusted OR: 2.1, CI: [1.1–3.9]) (Fig. 4). Similarly, individuals with already high trust in researchers from public organizations were more likely to report increased trust in the three types of public organizations if such website is implemented, compared with those with low trust (universities: adjusted OR: 2.6, CI: [1.4–4.8]; government-related organizations: adjusted OR: 3.9, CI: [2.1–7.3]; healthcare organizations: adjusted OR: 2.3, CI: [1.2–4.3]).

More than half of respondents indicated that their trust in researchers from private organizations, whether based in Quebec or outside Quebec, would increase if they had access to a secure website providing information on how their health data is used for research, with the strongest effect observed for researchers from Quebec companies (Table 11).

**Table 11:** Increased trust in researchers from private organizations with access to a secure website

Would a secure website increase your level of trust in researchers from:	Available data *n*/*N* (%)	Yes *n*/*N* (%)	No *n*/*N* (%)
Quebec private companies?	364 / 366 (99.5%)	244 / 364 (67.0%)	120 / 364 (33.0%)
Private companies based outside Quebec?	362 / 366 (98.9%)	192 / 362 (53.0%)	170 / 362 (47.0%)

**Fig. 5:**
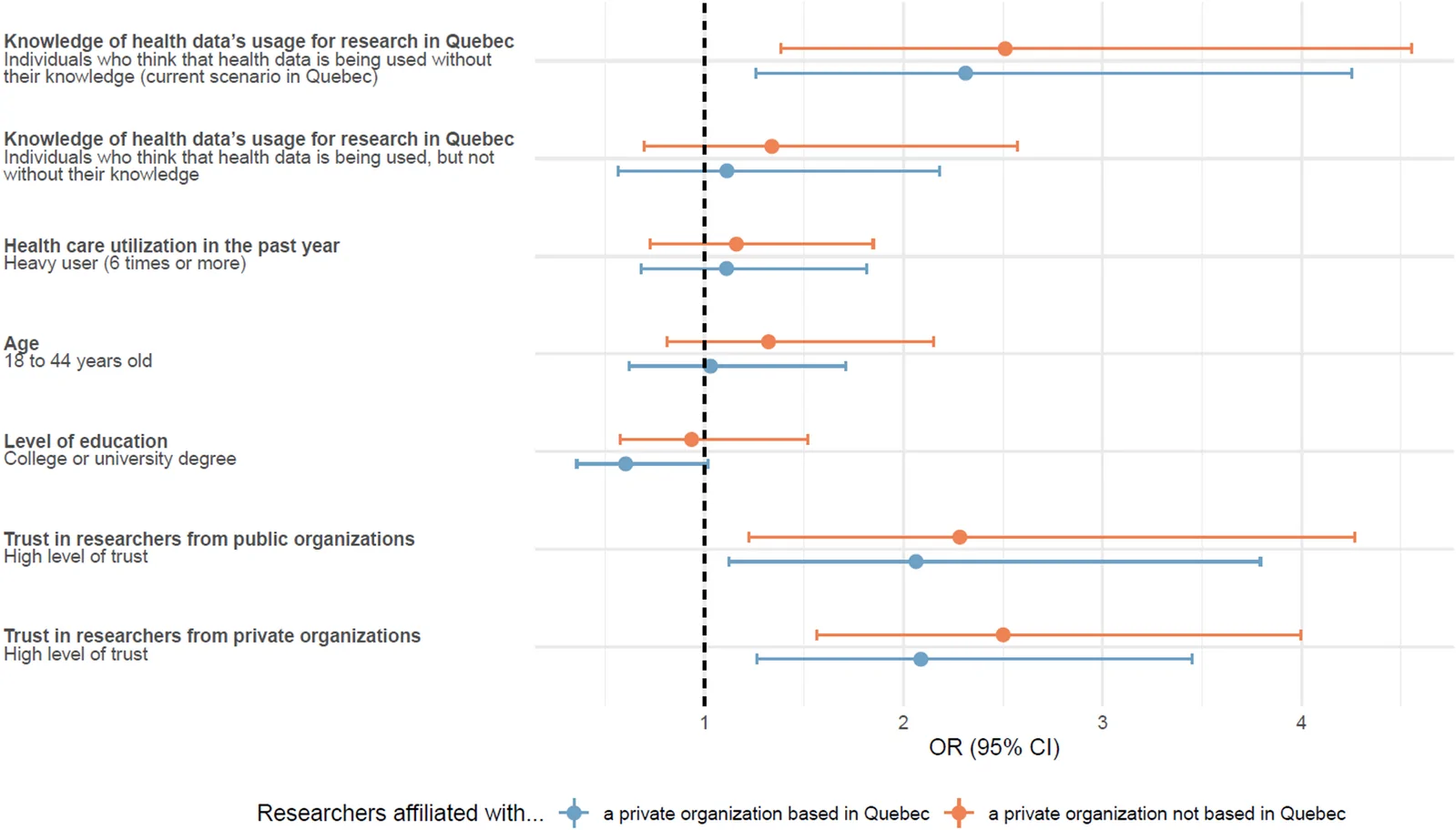
Predictors associated with increased trust in researchers from the two private organizations if a secure website is implemented

As observed with researchers from public organizations, individuals who are aware that their health data is currently used for research in Quebec without their knowledge were more likely to report increased trust in researchers from the two private organizations if a secure website is implemented, compared with those who do not believe their data is currently used (Quebec-based companies: adjusted OR: 2.3, CI: [1.3–4.3]; outside-Quebec companies: adjusted OR: 2.5, CI: [1.4–4.6]) (Fig. 5). Furthermore, individuals with high trust in researchers from public organizations were also more likely to experience increased trust in researchers from the two private organizations if a secure website is implemented, compared with those with low trust in researchers from public organizations (Quebec-based companies: adjusted OR: 2.1, CI: [1.1–3.8]; outside-Quebec companies: adjusted OR: 2.3, CI: [1.2–4.3]). Finally, individuals with high trust in researchers from private organizations themselves were more likely to report increased trust in these researchers if a secure website is implemented, compared with those with low trust (Quebec-based companies: adjusted OR: 2.1, CI: [1.3–3.5]; outside-Quebec companies: adjusted OR: 2.5, CI: [1.6-4.0]) (Fig. 5).

## Discussion

### Principal contributions

Our study offers important insights into public perceptions of transparency in the secondary use of health data for research. This study reaffirms that the role of secondary use of health data has not yet percolated to the general population. Prioritizing the development of a web-based patient portal to provide information about health data use would increase trust and meet citizen expectations regarding transparency. Although our sample was slightly younger and more literate than the general population, respondents showed comparable internet access, supporting the feasibility and relevance of a web‑based transparency portal.

Trust in public‑sector researchers remained high despite limited awareness of current data uses, while trust in private‑sector researchers was noticeably lower, reflecting concerns documented in prior literature. Our study also showed that transparency can meaningfully strengthen trust, particularly among younger adults, highlighting the need for tailored communication strategies that account for sociodemographic differences.

Selected exploratory analyses support a positive impact on trust even towards researchers from private organizations. Because there was an association between awareness of secondary use of health data and trust, providing more knowledge to a greater number of citizens represents an opportunity to create a virtuous cycle.

### Respondents’ awareness of the use of health data for research and trust towards researchers

More than half of respondents were unaware that their health data may be used for research purposes, confirming findings from previous studies [26–28]. Despite this lack of awareness, we observed high levels of trust in researchers from public organizations. In contrast, trust in researchers from private organizations was relatively low, a trend consistently reported in the literature. Public organizations generally benefit from greater public trust due to the collective benefits they provide and the non-commercial nature of their research activities. Conversely, private organizations tend to generate significant mistrust, often due to concerns about profit-driven motives, loss of control over the data, and a perceived lack of confidentiality and transparency [28–30].

Our results also reflect public perceptions of academic researchers and the attributes associated with trustworthiness [31], as well as the key role of social positioning in shaping relationships of trust [32]. When health data is used without individuals’ knowledge or consent, trustworthiness shifts from individuals to broader organizations and partnerships, entities whose trust cannot be taken for granted [33]. This reinforces the representativity of respondents regarding these trust and transparency questions.

### Transparency: impact on trust

Three-quarters of respondents indicated that their trust in research using their health data would increase if they were provided with information about such use. Interestingly, trust in public or private organizations did not emerge as a significant predictor of this increased trust. However, respondents’ age did. Individuals aged 18 to 44 were more likely to report increased trust if they were provided with information compared with those aged 45 or older. Other studies have also shown that variations in trust depend on sociodemographic characteristics, and that certain age groups, particularly younger individuals, are more likely to increase their trust when transparency efforts are implemented, whereas older individuals tend to show a less pronounced effect [28, 29, 34]. Some studies have further reported that older individuals express more privacy-related concerns than younger individuals [28].

This is an important aspect to consider for the eventual communication campaigns that would surround the launch of a web-based portal where communication strategies might differ based on the targeted age groups.

### Transparency mechanisms: priority for implementation

Our study also revealed that over 90% of respondents believe it is important to prioritize the implementation of provincial-wide transparency mechanisms in the coming years to better inform the population about how their health data is used for research purposes. Interestingly, awareness of current research uses of health data emerged as a predictor of the perceived importance of implementing such information mechanisms. This result aligns with previous findings in the literature: when individuals are aware that their health data may be used for research, they tend to express a stronger desire to receive clear and accessible information about these secondary uses, particularly regarding who accesses their data, the intended benefits, potential risks, and the safety measures in place [35, 36].

### Transparency mechanisms: a secure website as an acceptable approach

Over 75% of respondents identified the website as an effective way to be informed about the use of their health data for research, noting that most had internet access.

As mentioned earlier, our respondents underscored the importance of having access to information about how their health data is used for research. Once initial strategies are implemented, it will be essential to assess whether they effectively reach citizens with higher information needs and whether they succeed in increasing awareness, understanding, and trust. It will also be important not to assume that transparency will necessarily have positive impacts. Studies indicate that finding the right balance is essential to avoid overwhelming citizens, overusing public resources or inadvertently undermining trust [20].

Our survey also showed that such a website would positively influence trust in researchers from different types of public and private organizations. However, the individuals whose trust would be most impacted by the implementation of this website are those who are already aware of how health data is currently accessed for research in Quebec and those who already have high levels of trust in researchers from public organizations. The website would also have a positive impact on trust in researchers from private organizations among people who already trust this type of organization.

### Representativity

Overall, our study sample is comparable to the broader Quebec population in terms of sociodemographic characteristics. However, we observed that our respondents had higher education levels and tended to be slightly younger than the general population. At the same time, internet access was very similar to that of the broader population, which supports the relevance and potential usefulness of web‑based transparency mechanisms such as an online portal.

### Limitations

This study has some limitations. First, the sample size in this survey is small and cannot be considered representative of Quebec’s population. However, the goal of this survey was not to achieve representativeness, but rather identifying avenues for exploration in the focus groups planned as part of our research program.

In comparison with previous studies, the response rate for this telephone survey was lower (9.4%) [9]. Polling firms are increasingly favoring web platforms due to feasibility concerns. We opted for a telephone survey, despite its significant higher cost, in order to reach individuals with reading difficulties or lower numerical literacy as pooling exclusively individuals already familiar enough with the web to be part of pooling panels would have been introducing biases and the literature is unclear on the impact of web surveys for certain types of questions remains unclear [37–40].

We used factorial analyses to guide our choice of scenario to reduce dimensionality. Alternative analysis tools could have been used to achieve similar results, such as polychoric principal component analysis or multiple correspondence analysis.

For our regression analyses, we used multiple imputations to address missing values, based on the assumption that the data was missing at random, noting that one of the survey questions used as predictor had a percentage of missing information above 12%. While it is possible that the missing values were not missing at random, this assumption is inherently untestable and should be acknowledged as a limitation. Complete-case analyses presented in Supplementary Material 2 led to results similar to those obtained with multiple imputations.

## Conclusion

Our results reveal strong support for the short-term implementation of transparency mechanisms. A secure website was perceived as an acceptable method for providing information about the use of health data for research, and our findings suggest that it could enhance trust across different types of research organizations, especially among individuals who are already somewhat informed or trusting. Taken together, these findings underscore both the public demand and practical viability of developing a secure online transparency portal, while also emphasizing the value of initially targeting specific individual profiles in implementation efforts.

## Supplementary Information

Supplementary Material 1.

Supplementary Material 2.

## Data Availability

The datasets generated and analyzed during the current study are not publicly available due to privacy and ethical reasons. Study participants have not provided consent for their data to be shared publicly.

## Abbreviations

CI: Confidence Interval
OR: Odd ratio
N: Number
