# Supplementary material for: Operationalizing transparency regarding the use of health data for research: a cross-sectional survey of citizens’ perspectives
Source: BMC Public Health. 2026 Jul 8;26:2737. doi: 10.1186/s12889-026-28232-0 (PMC13628965; doi:10.1186/s12889-026-28232-0)
Supplement: Supplementary file 1 [file 12889_2026_28232_MOESM1_ESM.docx]

**Supplementary Material 1. Survey questionnaire**

/*Context */

Q_1NTRO	The purpose of the survey is to guide the implementation of concrete ways to better meet the transparency expectations of the Quebec population regarding research projects being done using health data.

In the survey, when we talk about health data, we are referring to, for example, your medical history, your medication list, your test results, your date of birth or your gender. It can be found in your files at the pharmacy, at your family doctor and at the hospital.

[Qknow1]	In Quebec, do you think your health data is used for research?

1=*Yes

2=*No

9=*Do not know/Do not answer

/*	*Only display sub-question [Qknow2] if the respondent answered “YES” to question [Qknow1]. */

[Qknow2]	In Quebec, do you think your health data is used for research without your knowledge?

1=*Yes

2=*No

9=*Do not know/Do not answer

[Qinfo]	You should know that health data is protected by Quebec laws. It may be used by researchers without your knowledge or permission, but only with the authorization of an ethics committee.

Using the health data of many people is important. Among other things, it allows researchers to make discoveries to improve care and services for the population.

The survey does not assess what you know about the subject. We want to know your opinion. Just answer as best you can.

/*Survey */

[Qpretrust-uni]	For the next 5 questions, we want to know your level of trust in researchers from various organizations who might use your health data for their research.

What level of trust do you have…

In researchers working at a university?

Would you say your level of trust is...

1=Very low

2=Low

3=High

4=Very high

9=*Do not know/Do not answer

[Qpretrust-gov]	In researchers working in a government-related organization? For example: The Quebec National Institute of Public Health.

Would you say your level of trust is...

1=Very low

2=Low

3=High

4=Very high

9=*Do not know/Do not answer

[Qpretrust-health]	In researchers working in the healthcare system? For example: a hospital.

Would you say your level of trust is...

1=Very low

2=Low

3=High

4=Very high

9=*Do not know/Do not answer

[Qpretrust-privQc]	In researchers working in a Quebec company? For example: who creates new drugs and medical devices in Quebec.

Would you say your level of trust is...

1=Very low

2=Low

3=High

4=Very high

9=*Do not know/Do not answer

[Qpretrust-privOut]	In researchers working in a company based outside Quebec? For example: who creates new drugs and medical devices outside Quebec.

Would you say your level of trust is...

1=Very low

2=Low

3=High

4=Very high

9=*Do not know/Do not answer

[Qinfo-trust]	Ways could be put in place to inform you about how your health data is used for research.

If you had access to information, would it increase your trust in the use of your health data for research?

1=*Yes

2=*No

9=*Do not know/Do not answer

[Qaccess-who]	If you had access to information, what level of importance would you give...

To know who gave permission to use your health data for a project when it was not you who gave it? For example, an ethics committee or a hospital director.

Would you say the level of importance is...

1=Very low

2=Low

3=High

4=Very high

9=*Do not know/Do not answer

[Qaccess-what]	To know what health data was used?

Would you say the level of importance is...

1=Very low

2=Low

3=High

4=Very high

9=*Do not know/Do not answer

[Qaccess-obj]	To know the objectives of the researchers who use your health data?

Would you say the level of importance is...

1=Very low

2=Low

3=High

4=Very high

9=*Do not know/Do not answer

[Qaccess-gen]	To be able to consult the results of research that used the health data from the general population?

Would you say the level of importance is...

1=Very low

2=Low

3=High

4=Very high

9=*Do not know/Do not answer

[Qaccess-own]	To be able to consult the results of research who used your own health data?

Would you say the level of importance is...

1=Very low

2=Low

3=High

4=Very high

9=*Do not know/Do not answer

[Qaccess-misuse]	To learn about the penalties for researchers in the event of misuse of your health data?

Would you say the level of importance is...

1=Very low

2=Low

3=High

4=Very high

9=*Do not know/Do not answer

[Qinfo-priority]	In the next 5 years, would it be important for Quebec citizens to have access to ways to inform them about how their health data is used for research?

1=*Yes

2=*No

9=*Do not know/Do not answer

[Qinfo-website]	In your opinion, would a secure website be a good way to inform you about how your health data is used for research?

1=*Yes

2=*No

9=*Do not know/Do not answer

[Qinctrust-uni]	Would such a secure website increase your level of trust…

In researchers working at a university?

1=*Yes

2=*No

9=*Do not know/Do not answer

[Qinctrust-gov]	In researchers working in a government-related organization? For example: The Quebec National Institute of Public Health.

1=*Yes

2=*No

9=*Do not know/Do not answer

[Qinctrust-health]	In researchers working in the healthcare system? For example: a hospital.

1=*Yes

2=*No

9=*Do not know/Do not answer

[Qinctrust-privQc]	In researchers working in a Quebec company? For example: who creates drugs and medical device in Quebec.

1=*Yes

2=*No

9=*Do not know/Do not answer

[Qinctrust-privOut]	In researchers working in a company based outside Quebec? For example: who creates drugs and medical device outside Quebec.

1=*Yes

2=*No

9=*Do not know/Do not answer

/*Respondent characteristics */

[SQsex]	What was your sex at birth?

Sex at birth refers to sex assigned at birth.

2=Female

1=Male

9=*Do not answer

[SQgenre]	What is your gender?

Gender refers to the gender with which you currently identify, which may be different from your sex at birth.

1=Male

2=Female

90=Other <specify>

99=*Do not answer

[SQage]	To which age group do you belong?

1=18-24 years old

2=25-34 years old

3=35-44 years old

4=45-54 years old

5=55-64 years old

6=65-74 years old

7=75 years old or over

9=*Do not answer

[SQedu]	What is the highest diploma you have obtained?

1=No certificate, diploma or degree (elementary school only or secondary school not completed)

2=Secondary school diploma or equivalent (attestation of equivalence)

3=Certificate or diploma from an apprenticeship program, a trade school or a vocational training centre (e.g., hairdressing, cooking, electrical trade, carpentry)

4=Certificate or diploma from a college, CEGEP or other non-university institution

5=University certificate, diploma or degree

90=Other

99=*Do not answer

[SQmotherlang]	What is your mother tongue, the language you first learned at home as a child and still understand?

*Multiple choices

*choicesminmax=1,3

1=English

2=French

90=Other <specify>

99=*Do not answer

[SQlanguage]	What language do you speak most often at home?

1=English

2=French

90=Other <specify>

99=*Do not answer

[SQcanada]	Were you born in Canada?

1=*Yes

2=*No

9=*Do not answer

[SQquebec]	How many years have you lived in Quebec?

This includes permanent or temporary residency.

1=Less than 5 years

2=5 years or more

9=*Do not answer

[SQgroup]	Do you belong to one of the following groups?

First Nations, Métis or Inuit?

1=*Yes

2=*No->SD9

9=*Do not answer->SD9

[SQgroup1]	Which one?

*multiple choices

*choiceminmax=1,3

1=First Nations (North American Indian)

2=Métis

3=Inuit

9=* Do not answer

[SQdis]	Person with disabilities?

* If necessary, specify: A person with a disability is someone who has a long term or recurring physical, mental, sensory, psychiatric or learning impairment and considers themselves to be disadvantaged in employment by reason of that impairment, and believes that an employer or potential employer is likely to consider them to be disadvantaged in employment by reason of that impairment. This definition also includes persons whose functional limitations owing to their impairment have been accommodated in their current job or workplace.

1=*Yes

2=*No

9=* Do not answer

[SQminority]	Member of visible minorities?

*If necessary, specify: A person in a visible minority group is someone, other than an Indigenous person, who is non-white in colour or race.

Here are a few examples of people who are part of a visible minority group within the meaning of the Equity Employment Act (please note that this list is not exhaustive):

- Black (African, Haitian, Jamaican, etc.);

- South Asian (Bengali, Tamil, Indian from India, etc.);

- Chinese (Hongkonger, Chinese, Mongolian, etc.);

- Korean;

- Japanese;

- Southeast Asian (Vietnamese, Cambodian, Thai, Laotian, etc.);

- Filipino;

- Pacific Islander;

- West Asian and Arab (Armenian, Iranian, Lebanese, Moroccan, Egyptian, Turk, etc.);

- Latin American (Brazilian, Colombian, Cuban, Peruvian, Guatemalan, etc.).*

1=*Yes

2=*No

9=*Do not answer

[SQhcu]	In the last 12 months, how often have you used physical or mental health services, either for yourself or to accompany a loved one? This also includes health services in private clinics, such as physiotherapists or psychologists.

1=0 times

2=1-2 times

3=3-5 times

4=6-12 times

5=More than 12 times

9=*Do not answer

[SQinternet]	Do you have regular access to the Internet?

*If necessary, specify: Internet at home, at work, at school or at the library through a computer or mobile device such as a smartphone or tablet.

1=*Yes

2=*No

9=*Do not know/Do not answer

[SQproject]	Are you currently participating, or have you participated in the past in a health research project?

*If necessary, specify: All participation in health research projects is relevant. For example, as a research subject, as a patient partner, etc.

1=*Yes

2=*No

9=*Do not know/Do not answer

/*END OF SURVEY*/
