# Supplementary material for: Operationalizing transparency regarding the use of health data for research: a cross-sectional survey of citizens’ perspectives
Source: BMC Public Health. 2026 Jul 8;26:2737. doi: 10.1186/s12889-026-28232-0 (PMC13628965; doi:10.1186/s12889-026-28232-0)
Supplement: Supplementary file 2 [file 12889_2026_28232_MOESM2_ESM.docx]

**Supplementary Material 2. Additional Methods and Results**

1. **Trust in researchers from different types of organizations**

As explained in the Variable derivation section of the manuscript, questions related to trust in five types of organizations, i.e. [Qpretrust-uni; Qpretrust-gov; Qpretrust-health; Qpretrust-privQc; Qpretrust-privOut], were used to derive one or multiple predictors. We recall that the three following scenarios were considered to account for potential correlations between the questions:

- Scenario A (5 variables). Trust in the five types of organizations [Qpretrust-uni; Qpretrust-gov; Qpretrust-health; Qpretrust-privQc; Qpretrust-privOut] has a minor correlation structure. Trust in each organization should be considered on its own and be used as a predictor.
- Scenario B (2 variables). Trust towards the three types of public organizations [Qpretrust-uni; Qpretrust-gov; Qpretrust-health] is highly correlated. A single variable combining this information could be used as a predictor [VarTrust-Pub]. Also, trust in the two types of private organizations [Qpretrust-privQc; Qpretrust-privOut] is highly correlated. A single variable combining this information could be used as a predictor [VarTrust-Priv].
- Scenario C (1 variable). Trust in the five types of organizations is highly correlated. A single variable combining this information could be used as a predictor.

In the following, we describe the methodology and results of the analysis conducted to choose the most adequate scenario. We also describe how the variables derived from the analysis were dichotomized.

1.1. **Dimensionality reduction for the regression models: Scenario A, B or C**

**Correlation matrix assessment**

The correlation matrix for the items [Qpretrust-uni; Qpretrust-gov; Qpretrust-health; Qpretrust-privQc; Qpretrust-privOut] was first assessed to determine whether it was reasonable to combine at least some of these items to reduce the dimensionality of the regression models, i.e. whether Scenario A could be discarded. More specifically, we checked if the correlation structure was weak, defined here as all pairwise absolute correlations between items being below 0.3, and considered that a weak correlation structure would indicate that the items should not be combined. The correlation matrices based on both the Spearman’s correlation and the polychoric correlation were examined, as the items were measured on a Likert scale and normality was therefore not expected to hold.

From the results presented in Tables S1 and S2, since all correlations were above 0.3, it was established that the correlation was not weak and Scenario A was therefore discarded.

Table S1: Spearman Correlation matrix

|  | Qpretrust-uni | Qpretrust-gov | Qpretrust-health | Qpretrust-privQc | Qpretrust-privOut |
| --- | --- | --- | --- | --- | --- |
| Qpretrust-uni | 1.0000000 | 0.6470622 | 0.5240225 | 0.4305222 | 0.3128935 |
| Qpretrust-gov | 0.6470622 | 1.0000000 | 0.5459826 | 0.4483518 | 0.3501112 |
| Qpretrust-health | 0.5240225 | 0.5459826 | 1.0000000 | 0.4083667 | 0.3314417 |
| Qpretrust-privQc | 0.4305222 | 0.4483518 | 0.4083667 | 1.0000000 | 0.6790693 |
| Qpretrust-privOut | 0.3128935 | 0.3501112 | 0.3314417 | 0.6790693 | 1.0000000 |

Table S2: Polychoric Correlation matrix

|  | Qpretrust-uni | Qpretrust-gov | Qpretrust-health | Qpretrust-privQc | Qpretrust-privOut |
| --- | --- | --- | --- | --- | --- |
| Qpretrust-uni | 1.0000000 | 0.7564605 | 0.6376713 | 0.5428630 | 0.3866525 |
| Qpretrust-gov | 0.7564605 | 1.0000000 | 0.6536839 | 0.5341464 | 0.4235250 |
| Qpretrust-health | 0.6376713 | 0.6536839 | 1.0000000 | 0.5147459 | 0.4110124 |
| Qpretrust-privQc | 0.5428630 | 0.5341464 | 0.5147459 | 1.0000000 | 0.7807643 |
| Qpretrust-privOut | 0.3866525 | 0.4235250 | 0.4110124 | 0.7807643 | 1.0000000 |

**Exploratory factor analysis**

As Scenario A was discarded following the assessment of the correlation matrix, we chose between Scenario B and Scenario C through exploratory factor analyses (EFA) [2]. EFAs were ran using R’s lavaan package (v.0.6.21). We used the weighted least square mean and variance adjusted estimator since all items are ordinal variables. Fit indices for this library and for this type of data is currently limited to a comparative fit index (CFI) and root mean square error of approximation (RMSEA).

Prior to conducting the EFA, the correlation structure was verified using a Bartlett’s test of sphericity. The presence of a non-weak correlation structure between items was reinforced by the Bartlett’s test of sphericity: we observed a small p-value (<0.001) and rejected the absence of correlation between items. We also verified if our data was suitable for an EFA by assessing the measures of sampling adequacy (MSA) of each item as well as the KMO, with values greater than 0.7 being considered acceptable [5].

Table S3: Measures of sampling adequacy

| Item | Qpretrust-uni | Qpretrust-gov | Qpretrust-health | Qpretrust-privQc | Qpretrust-privut | Global |
| --- | --- | --- | --- | --- | --- | --- |
| MSA | 0.78 | 0.79 | 0.89 | 0.72 | 0.68 |  |
| KMO |  |  |  |  |  | 0.77 |

As observed in Table S3, all measures but the one related to trust in private organizations outside Quebec can be considered acceptable. As there is a single item not meeting this criterion and its MSA is close to 0.7, we chose to keep all items in the model as is, which aligns with our aim of dimension reduction.

As a first step in the EFA, the number of factors to be included in the analysis was chosen using both the MAP test and a parallel analysis. The results of the MAP test are provided in Table S4 and the parallel analysis is illustrated in Figure S1.

Table S4: MAP values

| Model | MAP |
| --- | --- |
| 1-factor | 0.1779 |
| 2-factors | 0.1799 |
| 3-factors | 0.3987 |
| 4-factors | 1 |

Figure S1: Parallel analysis scree plots

The results of the MAP test suggested that a single factor should be retained, as it is the model associated with the smallest MAP value. However, the MAP value obtained for the 2-factors model was also very close to the one of the 1-factor model, and, from Figure S1, the parallel analysis suggested using two factors. Consequently, both the 1-factor and 2-factor models were compared. For the 2-factors model, an oblique (direct oblimin) factor rotation was used since trust towards public and private organizations, i.e. the expected interpretations of the underlying latent variables, might had been correlated.In the EFA, Scenario B was deemed adequate if, for every item of the same variable within the scenario, their strongest loading was observed within the same factor. Scenario C (only one factor) was deemed adequate if all factor loadings were of 0.5 or above.

For reference (see [3]), factor loadings of at least 0.72 are considered strong, whereas loadings of at least 0.32 but smaller than 0.71 are considered weak.

Table S5. Results of the EFA: Factor loadings and tests

| **Item** | **1-factor**  **(Scenario C)** | **2-factor**  **(Scenario B)** | |
| --- | --- | --- | --- |
|  | Factor 1 | Factor 1 | Factor 2 |
| [Qpretrust-uni] | **0.81** | **0.88** | -0.02 |
| [Qpretrust-gov] | **0.82** | **0.88** | -0.01 |
| [Qpretrust-health] | **0.75** | **0.69** | 0.09 |
| [Qpretrust-privQc] | **0.75** | 0.19 | **0.76** |
| [Qpretrust-privOut] | **0.63** | -0.08 | **0.95** |
|  |  |  |  |
| **p-value for the adjusted chi-square goodness of fit test** | <0.001 | 0.08 | |
|  |  |  | |
| **RMSEA** | 0.391 | 0.085 | |
| **CFI** | 0.735 | 0.997 | |

As shown in Table S5, all items had factor loadings greater than 0.5 (between 0.63 and 0.82) to the single factor of Scenario C. For Scenario B, [Qpretrust-uni; Qpretrust-gov; Qpretrust-health] all correlated the most to the first factor (loadings between 0.69 and 0.88), while [Qpretrust-privQc; Qpretrust-privOut] correlated the most to the second factor (loadings between 0.76 and 0.95). This supports the idea of public organization related items being correlated to each other, as well as private organization related items being correlated to each other.As both scenarios were deemed adequate, the adjusted chi-square goodness of fit test was used to select a final scenario. However, the adjusted chi-square test cannot confirm on its own whether we should choose Scenario B over Scenario C, as a two-factor solution doesn’t imply that items [Qpretrust-uni; Qpretrust-gov; Qpretrust-health] and items [Qpretrust-privQc; Qpretrust-privOut] each correlate on a given factor. Furthermore, this test is known to be sensitive to sample size, which emphasizes why it should be used alongside fit indices to choose between scenarios. The RMSEA and CFI were therefore additionally used to support the choice of scenario. To establish goodness of fit, commonly accepted threshold for the RMSEA and difference in RMSEA ($\Delta$RMSEA) are below 0.08 and above 0.015 respectively, while commonly accepted threshold for the CFI and difference in CFI ($\Delta$CFI) are above 0.95 and above 0.01 respectively [6].

All results were presented in Table S5. The p-value for the chi-square goodness of fit test was very small for Scenario C, suggesting that the associated number of factors was inadequate. The p-value for the chi-square goodness of fit test was greater than 0.05 for Scenario B, suggesting that the associated number of factors was appropriate. The RMSEA for Scenario C was 0.391, which suggested a bad fit, while this index was on the borderline of acceptance for Scenario B with a value of 0.085, showing an improvement on Scenario C ($\Delta$RMSEA = 0.306). The CFI for Scenario C was 0.735, which also suggested a bad fit, whereas the same index for Scenario B indicated a good fit with a value of 0.997, again showing improvement on Scenario C ($\Delta$CFI = 0.262).

In conclusion, the results of the EFA led to the choice of Scenario B. Two binary variables were therefore created using the five trust-related items, as detailed in the next section.

**Confirmatory factor analysis**

We also tried choosing between scenarios using confirmatory factor analyses. However, fitting the model of Scenario B proved to be a challenge, as the trust towards private organizations factor was measured by only 2 items. This CFA led to a Heywood case, where we observed both a negative residual variance for the trust towards private organizations in Quebec as well as a standardized factor loading greater than 1 for that same item. Constraining either the loading or the residual variance did not fix this issue, but fixing equal loadings for the items on the second factor resulted in a valid model. However, we had no theoretical rationale for the equal loading assumption: it could be that one type of organization weighs in more than another (e.g. private organizations of Quebec vs in Quebec). As such, this model was not considered and CFA was deemed unsuitable.

1.2. **Dichotomization of the variable**

Trust in researchers from public institutions was defined as a binary variable (low vs. high) based on responses to question [Qpretrust-uni; Qpretrust-gov; Qpretrust-health], which evaluated trust in researchers from a university, a government-related organization, and the healthcare system, respectively. Similarly, trust in researchers from private institutions was defined as a binary variable (low vs. high) based on responses to items [Qpretrust-privQc; Qpretrust-privOut], which assessed trust in researchers from private organizations based in Quebec and outside Quebec, respectively. Details are given in Table S6.

Table S6. Dichotomization of the variable for trust in researchers

| Trust profile | Question in the survey | Details on the profile creation |
| --- | --- | --- |
| [VarTrust-Pub] | [Qpretrust-uni]  [Qpretrust-gov] [Qpretrust-health] | Initial trust scale: Very low=1; Low=2; High=3; Very high=4.  If the answer is 1 (very low) to at least one of the three questions: The respondent is considered to have low trust (group 0).  Otherwise: Sum the answered questions divided by the number of answered questions (average score) and associate the group as follows:  Group 1. Average score > 2.5 (respondent is considered to have high trust)  Group 0. Average score ≤ 2.5 (respondent is considered to have low trust)  Note: If a respondent did not answer any of the three questions, their profile is considered a missing value. |
| [VarTrust-Priv] | [Qpretrust-privQc]  [Qpretrust-privOut] | Initial trust scale: Very low=1; Low=2; High=3; Very high=4.  If the answer is 1 (very low) to at least one of the two questions: The respondent is considered to have low trust (group 0).  Otherwise: Sum the answered questions divided by the number of answered questions (average score) and associate the group as follows:  Group 1. Average score > 2.5 (respondent is considered to have high trust)  Group 0. Average score ≤ 2.5 (respondent is considered to have low trust)  Note: If a respondent did not answer any of the two questions, their profile is considered a missing value. |

2. **Regression Analyses**
   2.1. **Predictors and outcomes**

Table S7. Description of predictors and outcomes for regression analyses

| Predictors | Categorization |
| --- | --- |
| [VarKnow] | Individuals who don’t think that their health data is being used for research* |
|  | Individuals who think that their health data is being used for research, but not without their knowledge |
|  | Individuals who think that their health data is being used for research without their knowledge |
| [VarHcu] | Light user (5 times or less)* |
|  | Heavy user (6 times or more) |
| [VarAge] | 18 to 44 years old |
|  | 45 years old or more* |
| [VarEdu] | No degree, high school degree or vocational degree* |
|  | College or university degree |
| [VarTrust-Pub] | Low level of trust* |
|  | High level of trust |
| [VarTrust-Priv] | Low level of trust* |
|  | High level of trust |
| *Reference group | |

| Outcomes | Categorization |
| --- | --- |
| [Qinfo-trust] | Yes |
|  | No* |
| [Qinfo-priority] | Yes |
|  | No* |
| [Qinfo-website] | Yes |
|  | No* |
| [Qinctrust-uni,-gov,-health,-privQC,-privOut] | Yes |
|  | No* |
| [VarTrust-Pub] (Sensitivity analysis) | Low level of trust* |
|  | High level of trust |
| [VarTrust-Priv] (Sensitivity analysis) | Low level of trust* |
|  | High level of trust |

2.2. **Model definitions**

Ten logistic regressions were used with respectively each single outcome listed in the previous section as response variables. For each model, all predictors defined in the previous section, excluding the response variable to avoid perfect collinearity, were used.

The models with [VarTrust-Pub] and [VarTrust-Priv] as response variables were used to identify any potential mediator variable, whereas the eight other models were used to look for associations between the main study outcomes and [Qinctrust] with any of the given predictors.

2.3. **Multiple imputation**

Multiple imputation by chained equations [6] was used to address missing values before creating the regression models described above, based on the assumption that the data was missing at random. Only variables included in the regression models were used in the imputation procedure (predictors and study outcomes). All variables were imputed using a logistic regression. A total of 25 datasets were created and Rubin’s Rules were used to derive coefficients estimates, confidence intervals as well as p-values. In a sensitivity analysis, the results obtained using multiple imputations were compared with those obtained through complete-case analysis.

2.4. **Detailed results and sensitivity analysis results**

**Exploratory regression analysis: Multiple Imputation and Complete-case results**

All results obtained for the exploratory regression analysis using multiple imputation were compared to the results obtained using complete-cases analyses. The results shown thereafter in Tables S8-S15 detail the comparison for using complete-case analysis and using multiple imputation for the logistic regression models discussed in the section Exploratory regression-based analyses in the main article.

Table S8. Regression analysis results for [Qinfo-trust] as outcome

| **[Qinfo-trust]** | **Multiple imputation** | | | **Complete case** | | |
| --- | --- | --- | --- | --- | --- | --- |
| **Predictors** | **OR** | **95% CI** | **p-value** | **OR** | **95% CI** | **p-value** |
| **[VarKnow]** |  |  |  |  |  |  |
| 1 | — | — |  | — | — |  |
| 2 | 0.76 | 0.42, 1.39 | 0.4 | 0.76 | 0.41, 1.42 | 0.4 |
| 3 | 0.84 | 0.40, 1.75 | 0.6 | 0.84 | 0.41, 1.81 | 0.6 |
| **[VarHcu]** | 1.09 | 0.64, 1.85 | 0.7 | 1.16 | 0.66, 2.07 | 0.6 |
| **[VarAge]** | 1.98 | 1.10, 3.57 | **0.023** | 2.26 | 1.21, 4.48 | **0.014** |
| **[VarEdu]** | 0.81 | 0.47, 1.41 | 0.5 | 0.83 | 0.46, 1.48 | 0.5 |
| **[VarTrust-Pub]** | 1.71 | 0.89, 3.28 | 0.11 | 1.35 | 0.65, 2.74 | 0.4 |
| **[VarTrust-Priv]** | 1.02 | 0.60, 1.74 | >0.9 | 0.96 | 0.54, 1.72 | 0.9 |
| Abbreviations: CI = Confidence Interval, OR = Odds Ratio | | | | | |  |

Table S9. Regression analysis results for [Qinfo-priority] as outcome

| **[Qinfo-priority]** | **Multiple imputation** | | | **Complete case** | | |
| --- | --- | --- | --- | --- | --- | --- |
| **Predictors** | **OR** | **95% CI** | **p-value** | **OR** | **95% CI** | **p-value** |
| **[VarKnow]** |  |  |  |  |  |  |
| 1 | — | — |  | — | — |  |
| 2 | 4.63 | 1.33, 16.2 | **0.016** | 4.04 | 1.32, 17.6 | **0.029** |
| 3 | 1.24 | 0.47, 3.30 | 0.7 | 1.19 | 0.47, 3.46 | 0.7 |
| **[VarHcu]** | 0.63 | 0.29, 1.35 | 0.2 | 0.63 | 0.28, 1.40 | 0.2 |
| **[VarAge]** | 1.00 | 0.45, 2.26 | >0.9 | 1.06 | 0.46, 2.61 | 0.9 |
| **[VarEdu]** | 0.82 | 0.35, 1.93 | 0.7 | 0.78 | 0.31, 1.84 | 0.6 |
| **[VarTrust-Pub]** | 1.25 | 0.45, 3.47 | 0.7 | 0.84 | 0.23, 2.49 | 0.8 |
| **[VarTrust-Priv]** | 1.18 | 0.53, 2.60 | 0.7 | 1.06 | 0.47, 2.44 | 0.9 |
| Abbreviations: CI = Confidence Interval, OR = Odds Ratio | | | | | | |

Table S10. Regression analysis results for [Qinfo-website] as outcome

| **[Qinfo-website]** | **Multiple imputation** | | | **Complete case** | | |
| --- | --- | --- | --- | --- | --- | --- |
| **Predictors** | **OR** | **95% CI** | **p-value** | **OR** | **95% CI** | **p-value** |
| **[VarKnow]** |  |  |  |  |  |  |
| 1 | — | — |  | — | — |  |
| 2 | 1.84 | 0.97, 3.48 | 0.060 | 1.80 | 0.94, 3.60 | 0.084 |
| 3 | 0.87 | 0.44, 1.75 | 0.7 | 0.88 | 0.44, 1.81 | 0.7 |
| **[VarHcu]** | 1.33 | 0.77, 2.28 | 0.3 | 1.36 | 0.77, 2.45 | 0.3 |
| **[VarAge]** | 2.05 | 1.13, 3.71 | **0.018** | 1.98 | 1.07, 3.83 | **0.034** |
| **[VarEdu]** | 1.05 | 0.61, 1.79 | 0.9 | 1.10 | 0.62, 1.94 | 0.7 |
| **[VarTrust-Pub]** | 2.06 | 1.08, 3.94 | **0.028** | 1.98 | 0.98, 3.96 | 0.055 |
| **[VarTrust-Priv]** | 0.96 | 0.57, 1.63 | 0.9 | 0.89 | 0.50, 1.58 | 0.7 |
| Abbreviations: CI = Confidence Interval, OR = Odds Ratio | | | | | | |

Table S11. Regression analysis results for [Qinctrust-uni] as outcome

| **[Qinctrust-uni]** | **Multiple imputation** | | | **Complete case** | | |
| --- | --- | --- | --- | --- | --- | --- |
| **Predictors** | **OR** | **95% CI** | **p-value** | **OR** | **95% CI** | **p-value** |
| **[VarKnow]** |  |  |  |  |  |  |
| 1 | — | — |  | — | — |  |
| 2 | 1.54 | 0.86, 2.76 | 0.15 | 1.49 | 0.82, 2.78 | 0.2 |
| 3 | 1.05 | 0.52, 2.11 | 0.9 | 1.03 | 0.53, 2.09 | >0.9 |
| **[VarHcu]** | 0.79 | 0.48, 1.28 | 0.3 | 0.74 | 0.44, 1.26 | 0.3 |
| **[VarAge]** | 1.67 | 0.99, 2.83 | 0.056 | 1.47 | 0.84, 2.63 | 0.2 |
| **[VarEdu]** | 0.66 | 0.39, 1.12 | 0.12 | 0.68 | 0.38, 1.18 | 0.2 |
| **[VarTrust-Pub]** | 2.60 | 1.40, 4.83 | **0.003** | 2.55 | 1.31, 4.99 | **0.006** |
| **[VarTrust-Priv]** | 0.85 | 0.52, 1.39 | 0.5 | 0.82 | 0.48, 1.41 | 0.5 |
| Abbreviations: CI = Confidence Interval, OR = Odds Ratio | | | | | | |

Table S12. Regression analysis results for [Qinctrust-gov] as outcome

| **[Qinctrust-gov]** | **Multiple imputation** | | | **Complete case** | | |
| --- | --- | --- | --- | --- | --- | --- |
| **Predictors** | **OR** | **95% CI** | **p-value** | **OR** | **95% CI** | **p-value** |
| **[VarKnow]** |  |  |  |  |  |  |
| 1 | — | — |  | — | — |  |
| 2 | 2.02 | 1.09, 3.74 | **0.026** | 2.31 | 1.24, 4.50 | **0.011** |
| 3 | 1.02 | 0.52, 2.00 | >0.9 | 1.04 | 0.53, 2.09 | >0.9 |
| **[VarHcu]** | 0.77 | 0.47, 1.27 | 0.3 | 0.70 | 0.41, 1.19 | 0.2 |
| **[VarAge]** | 1.30 | 0.78, 2.19 | 0.3 | 1.26 | 0.72, 2.25 | 0.4 |
| **[VarEdu]** | 0.92 | 0.55, 1.55 | 0.8 | 0.93 | 0.53, 1.61 | 0.8 |
| **[VarTrust-Pub]** | 3.90 | 2.10, 7.25 | **<0.001** | 3.80 | 1.96, 7.50 | **<0.001** |
| **[VarTrust-Priv]** | 1.18 | 0.72, 1.95 | 0.5 | 1.07 | 0.62, 1.86 | 0.8 |
| Abbreviations: CI = Confidence Interval, OR = Odds Ratio | | | | | | |

Table S13. Regression analysis results for [Qinctrust-health] as outcome

| **[Qinctrust-health]** | **Multiple imputation** | | | **Complete case** | | |
| --- | --- | --- | --- | --- | --- | --- |
| **Predictors** | **OR** | **95% CI** | **p-value** | **OR** | **95% CI** | **p-value** |
| **[VarKnow]** |  |  |  |  |  |  |
| 1 | — | — |  | — | — |  |
| 2 | 2.07 | 1.10, 3.89 | **0.024** | 2.05 | 1.07, 4.11 | **0.036** |
| 3 | 1.53 | 0.72, 3.24 | 0.3 | 1.52 | 0.73, 3.37 | 0.3 |
| **[VarHcu]** | 0.81 | 0.48, 1.35 | 0.4 | 0.76 | 0.43, 1.33 | 0.3 |
| **[VarAge]** | 1.14 | 0.66, 1.95 | 0.6 | 0.89 | 0.50, 1.60 | 0.7 |
| **[VarEdu]** | 0.62 | 0.35, 1.08 | 0.094 | 0.59 | 0.31, 1.09 | 0.10 |
| **[VarTrust-Pub]** | 2.30 | 1.21, 4.35 | **0.011** | 2.21 | 1.09, 4.45 | **0.026** |
| **[VarTrust-Priv]** | 1.09 | 0.64, 1.84 | 0.8 | 1.03 | 0.58, 1.86 | >0.9 |
| Abbreviations: CI = Confidence Interval, OR = Odds Ratio | | | | | | |

Table S14. Regression analysis results for [Qinctrust-privQc] as outcome

| **[Qinctrust-privQC]** | **Multiple imputation** | | | **Complete case** | | |
| --- | --- | --- | --- | --- | --- | --- |
| **Predictors** | **OR** | **95% CI** | **p-value** | **OR** | **95% CI** | **p-value** |
| **[VarKnow]** |  |  |  |  |  |  |
| 1 | — | — |  | — | — |  |
| 2 | 2.31 | 1.25, 4.27 | **0.008** | 2.29 | 1.24, 4.37 | **0.009** |
| 3 | 1.11 | 0.57, 2.19 | 0.8 | 1.12 | 0.57, 2.24 | 0.7 |
| **[VarHcu]** | 1.11 | 0.68, 1.82 | 0.7 | 1.01 | 0.59, 1.72 | >0.9 |
| **[VarAge]** | 1.03 | 0.62, 1.71 | >0.9 | 0.94 | 0.55, 1.64 | 0.8 |
| **[VarEdu]** | 0.60 | 0.36, 1.02 | 0.060 | 0.65 | 0.36, 1.13 | 0.13 |
| **[VarTrust-Pub]** | 2.06 | 1.12, 3.80 | **0.020** | 1.92 | 0.99, 3.72 | 0.051 |
| **[VarTrust-Priv]** | 2.09 | 1.26, 3.45 | **0.004** | 2.21 | 1.28, 3.89 | **0.005** |
| Abbreviations: CI = Confidence Interval, OR = Odds Ratio | | | |  |  |  |

Table S15. Regression analysis results for [Qinctrust-privOut] as outcome

| **[Qinctrust-privOut]** | **Multiple imputation** | | | **Complete case** | | |
| --- | --- | --- | --- | --- | --- | --- |
| **Predictors** | **OR** | **95% CI** | **p-value** | **OR** | **95% CI** | **p-value** |
| **[VarKnow]** |  |  |  |  |  |  |
| 1 | — | — |  | — | — |  |
| 2 | 2.51 | 1.38, 4.57 | **0.003** | 2.44 | 1.38, 4.40 | **0.002** |
| 3 | 1.34 | 0.69, 2.58 | 0.4 | 1.33 | 0.70, 2.56 | 0.4 |
| **[VarHcu]** | 1.16 | 0.73, 1.85 | 0.5 | 1.18 | 0.71, 1.95 | 0.5 |
| **[VarAge]** | 1.32 | 0.81, 2.16 | 0.3 | 1.34 | 0.79, 2.29 | 0.3 |
| **[VarEdu]** | 0.93 | 0.57, 1.52 | 0.8 | 0.93 | 0.55, 1.57 | 0.8 |
| **[VarTrust-Pub]** | 2.28 | 1.22, 4.28 | **0.010** | 2.37 | 1.22, 4.73 | **0.012** |
| **[VarTrust-Priv]** | 2.50 | 1.56, 4.00 | **<0.001** | 2.59 | 1.56, 4.36 | **<0.001** |
| Abbreviations: CI = Confidence Interval, OR = Odds Ratio | | | | | |  |

**Sensitivity analysis: Trust in public and private institutions**

Table S16. Regression analysis results for [VarTrust-Pub] as outcome

| **Characteristic** | **OR** | **95% CI** | **p-value** |
| --- | --- | --- | --- |
| **[VarKnow]** |  |  |  |
| 1 | — | — |  |
| 2 | 0.84 | 0.42, 1.68 | 0.6 |
| 3 | 2.95 | 1.04, 10.7 | 0.062 |
| **[VarHcu]** | 1.85 | 0.94, 3.79 | 0.083 |
| **[VarAge]** | 0.64 | 0.33, 1.27 | 0.2 |
| **[VarEdu]** | 2.43 | 1.27, 4.68 | **0.008** |
| **[VarTrust-Priv]** | 6.36 | 2.87, 16.2 | **<0.001** |
| Abbreviations: CI = Confidence Interval, OR = Odds Ratio | | | |

Table S17. Regression analysis results for [VarTrust-Priv] as outcome

| **Characteristic** | **OR** | **95% CI** | **p-value** |
| --- | --- | --- | --- |
| **[VarKnow]** |  |  |  |
| 1 | — | — |  |
| 2 | 0.72 | 0.40, 1.27 | 0.3 |
| 3 | 1.31 | 0.70, 2.47 | 0.4 |
| **[VarHcu]** | 0.87 | 0.52, 1.42 | 0.6 |
| **[VarAge]** | 1.23 | 0.73, 2.09 | 0.4 |
| **[VarEdu]** | 0.67 | 0.40, 1.13 | 0.14 |
| **[VarTrust-Pub]** | 6.40 | 2.89, 16.4 | **<0.001** |
| Abbreviations: CI = Confidence Interval, OR = Odds Ratio | | | |

**References for supplementary material 2**

1. Timothy A Brown. Confirmatory Factor Analysis for Applied Research. Methodology in the Social Sciences. Guilford Publications, 2015.
2. Fabrigar LR, Wegener DT. Exploratory factor analysis. Oxford: Oxford University Press; 2012.
3. Costello AB, Osborne J. Best practices in exploratory factor analysis: four recommendations for getting the most from your analysis. Practical Assessment, Research, and Evaluation. 2005; 10(1): 7.
4. van Buuren S, Groothuis-Oudshoorn K. mice: Multivariate Imputation by Chained Equations in R. J. Stat. Soft. 2011 Dec. 12;45(3):1-67.
5. Kaiser, H. F. (1974). An index of factorial simplicity. *psychometrika*, *39*(1), 31-36.
6. Finch, W. H. (2020). Using fit statistic differences to determine the optimal number of factors to retain in an exploratory factor analysis. *Educational and psychological measurement*, *80*(2), 217-241.
